# Safety and efficacy of cerebral embolic protection devices during TAVI: a meta-analysis and sequence analysis of trials

**DOI:** 10.1055/s-0046-1829157

**Published:** 2026-11-06

**Authors:** Aldo José Silva de Castro Costa, Marcus Vinícius Costa Fernandes, Thaynar Cavalcante Batista, Ana Virgínia de Jesus Félix, Tamara Larissa Silva Barbosa, Isis Maria Lima Cruz, Gustavo Sousa Noleto

**Affiliations:** 1 Universidade Federal do Piauí, Faculdade de MedicinaTeresina PIBrazil; 2 Universidade Federal do Piauí, Faculdade de Medicina, Hospital UniversitárioTeresina PIBrazil

**Keywords:** Embolic Protection Devices, Ischemic Stroke, Transcatheter Aortic Valve Replacement, Aortic Valve Stenosis

## Abstract

**Background:**

Cerebral embolic protection (CEP) devices have been developed to reduce postoperative neurological outcomes after transcatheter aortic valve implantation (TAVI); however, their effectiveness remains controversial.

**Objective:**

To evaluate the impact of CEP use during TAVI on the occurrence in clinical outcomes.

**Methods:**

Databases were used to search for randomized clinical trials. The occurrence of all strokes, of disabling strokes specifically, transient ischemic attacks (TIAs), all-cause mortality, and vascular complications were analyzed. Conventional meta-analysis was conducted using fixed-effects modeling and trial sequential analysis (TSA: type I error of 5%, power of 90%). The systematic review protocol was registered on the International Prospective Register of Systematic Reviews (PROSPERO; CRD420251081075).

**Results:**

In total, eight clinical trials were included, gathering 11,589 patients randomized between those who used the devices (5,943) and those who did not (5,646). The meta-analysis demonstrated no significant association between CEP use and reduced odds of all-cause stroke (OR = 0.92, 95% CI: 0.73–1.15, *p* = 0.46) and disabling stroke (OR = 0.80, 95% CI: 0.57–1.13, *p* = 0.20). The sequential analysis identified a lack of evidence for all strokes (required sample size [rSS] = 38,626; 10.53%), disabling strokes (rSS = 59,732; 17.05%). In terms of mortality, there was no significance in the use of devices (OR = 1.09, 95% CI: 0.71–1.69, *p* = 0.69), reaching evidence for support (rSS = 7,053; 42.25%);

**Conclusion:**

The data on the use of CEP is insufficient to support meta-analytic conclusions regarding cerebrovascular outcomes. Nevertheless, TSA demonstrated evidence supporting futility regarding mortality reduction.

## INTRODUCTION

In aortic stenosis, transcatheter aortic valve replacement (TAVI) has become a well-established option for patients with varying levels of surgical risk. However, TAVI still presents significant complications, such as the risk of periprocedural stroke, primarily due to embolization of valve or vascular debris. The occurrence of stroke is associated with prolonged hospitalization, functional deterioration, a 2-fold increase in readmission costs, and up to a 6-fold increase in 30-day mortality. 1 2

Cerebral embolic protection (CEP) devices are designed to capture debris released during TAVI and prevent it from reaching the brain. Initial studies have demonstrated debris recovery by filters, a reduction in the volume of new brain lesions on MRI, and potential benefits in smaller clinical trials. Despite this, the effectiveness of these devices remains under debate. 3

This study aims to evaluate the impact of the use of CEP devices during TAVI on the occurrence of cerebrovascular events (total strokes, disabling strokes, transient ischemic attacks [TIAs], and vascular complications) and mortality, compared with the procedure performed without the use of these devices.

## METHODS

### Study protocol

This study followed the guidelines contained in the Preferred Reporting Items for Systematic Reviews and Meta-Analyses (PRISMA) protocol and the guidelines contained in the *Cochrane Handbook for Systematic Reviews of Interventions* . 4 5 The systematic review protocol was registered on the International Prospective Register of Systematic Reviews (PROSPERO; CRD420251081075).

The Ethics Committee of Universidade Federal do Piauí waived the need for ethics approval and patient consent for the collection, analysis, and publication of the retrospectively obtained and anonymized, publicly available data for this non-interventional study.

### Search strategy

The study used the following databases during the search stage: Cochrane Library, Embase, PubMed/Medline, and ClinicalTrials; the search was not limited to the publication date or languages during this phase. The search strategy was performed on May 5, 2025, consisting of descriptors based on the Medical Subject Headings (MeSH) platform and related to the Boolean operators “AND” and “OR”: ( *Cerebral Embolic Protection* OR *Embolic Protection Devices* ) AND ( *Transcatheter Aortic Valve Replacement* OR *Transcatheter Aortic-Valve Implantation* ) AND *Stroke* .

### Study screening and selection

The studies identified during the search stage were exported to the Rayyan platform (Rayyan Systems Inc.), where duplicates were detected, manually removed, and the remaining articles were screened. This stage was performed independently and blindly by two researchers. If there were significant discrepancies between the judgments, a third evaluator was included. The screening was divided into two subphases: a preliminary assessment using the titles and abstracts of the articles according to the eligibility criteria; then, the remaining articles were inspected using the full text.

Studies that met the following criteria were included: blinded randomized clinical trials; studies involving individuals eligible for TAVI; use of CEP as the tested intervention; use of only transcatheter therapy in the control group; studies that analyzed the occurrence of total and disabling stroke as the efficacy outcome; a follow-up time of at least 72 hours or until the patient's hospital discharge after implantation.

Cohen's Kappa index was performed at the end of the screening. This metric results from a statistical analysis that considers the level of agreement and reliability in the judgment between two evaluators during the screening of the articles. The classification proposed by McHugh was considered for the interpretation of this statistical indicator. 6

### Data extraction and quality analysis

Data extraction was performed independently by two researchers using a predefined form in Excel (Microsoft Corp.). Finally, the extracted data were compared, and, if necessary, disagreements were resolved by a third researcher. The predefined spreadsheet for data removal is found in **Supplementary Material 1 Table S1** (available at https://www.arquivosdeneuropsiquiatria.org/wp-content/uploads/2026/09/ANP-2025.0503-Supplementary-Material-1-atualizado.docx ).

**Table 1:** Characteristics of the included randomized clinical trials

Study	Author/ year	Type of CEP device	Puncture site	Total (Int./Cont.)	Int. /Cont., n (%)	Male, n (%) (Int./Cont.)	Age ^€^ (Int./Cont.)	EuroSCORE II ^€¥^ (Int./Cont.)	Previous stroke (Int./Cont.)	Previous TIA (Int./Cont.)	Atrial fibrillation (Int./Cont.)	DM II (Int./Cont.)	Hypertension (Int./Cont.)
CLEAN-TAVI	Haussig et al., 2016 10	Claret Medical	Transfemoral	100	50 (50%) / 50 (50%)	21 (42%) / 22 (44%)	80 ± 5.1 / 79.3 ± 4.1	16.4 ± 10% / 14.5 ± 8.7%	1 (2%) / 3 (6%)¶		17 (34%) / 17 (34%)	20 (40%) / 25 (50%)	44 (88%) / 47 (94%)
SENTINEL	Kapadia et al., 2017 11	Sentinel	Radial and brachial artery	363	244 (67.2%) / 119 (32.8%)*	113 (46.3%) / 61 (51.3%)	83.1 (77.2–87.2) / 85.0 (78.4–89.4)	NDA	5 (4.1%) / 6 (5%)	9 (7.4%) / 8 (6.7%)	42 (34.7%) / 36 (30.3%)	49 (40.5%) / 45 (37.8%)	NDA
PROTECTED-TAVR	Kapadia et al., 2022 2	Sentinel	Transfemoral	3,000	1501 (50%) / 1499 (50%)	870 (58%) / 933 (62.2%)	78.9 ± 8 / 78.9 ± 7.8	4.6 ± 5.1 / 4.3 ± 5.2	114 (7.6%) / 122 (8.5%)	77 (5.2%) / 81 (5.4%)	511 (34.1%) / 469 (31.4%)	501 (33.4%) / 522 (34.8%)	1306 (87.1%) / 1312 (87.6%)
BHF-PROTECT-TAVR	Kharbanda et al., 2025 12	Sentinel	Radial artery	7,635	3798 (49.7%) / 3803 (50.3%)	2314 (60.9%) / 2342 (61.6%)	81.2 ± 6.5 / 81.3 ± 6.5	2.4 (1.6–4.1) / 2.4 (1.6–4.0)	217 (5.8%) / 235 (6.3%)	319 (8.5%) / 291 (7.8%)	1256 (33.5%) / 1269 (33.8%)	793 (20.9%) / 767 (20.2%)	2558 (68.4%) / 2528 (67.4%)
DEFLECT III	Lansky et al., 2015 13	TriGuard	Transfemoral	85	46 (54.1%) / 39 (45.9%)	20 (43.5%) / 19 (48.7%)	82.5 ± 6.5 / 82.3 ± 6.0	10.1 ± 10.1/ 7.2 ± 6.6	6 (13.3%) / 7 (17.9%)	NDA	10 (21.7%) / 14 (35.9%)	10 (21.7%) / 9 (23.1%)	37 (80.4 %) / 28 (71.8%)
REFLECT I	Lansky et al., 2021 14	TriGuard	Transfemoral	204	141 (69.1%) / 63 (30.9%)	80 (56.7%) / 42 (66.7%)	79.8 ± 7.3 / 81.5 ± 7.1	4.8 ± 4.1/ 5.5 ± 4.1	10 (7.5%) / 4 (6.7%)	10 (7.7%) / 4 (6.7%)	45 (33.1%) / 16 (25.8%)	60 (42.9%) / 20 (31.7%)	NDA
REFLECT II	Nazif et al., 2021 15	TriGuard	Transfemoral	179	157 (67.6%) / 58 (32.4%)	62 (55.4 %) / 35 (61.4 %)	79.71 ± 7.96 / 78.05 ± 8.19	3.69 ± 2.96 / 3.59 ± 3.41	12 (10.7 %) / 2 (3.5%)	9 (8.3%) / 2 (3.5 %)	32 (28.6%) / 17 (29.8%)	39 (34.8 %) / 23 (40.4%)	NDA
MISTRAL-C	Van Mieghem et al., 2016 16	Sentinel	Transfemoral	65	32 (49.2%) / 33 (50.8%)	17 (53.1%) / 17 (51.5)	82 (79–84) / 82 (77–86)	NDA	6 (19%) / 6 (18%)		8 (29%) / 8 (27%)	4 (13%) / 9 (27%)	21 (66%) / 23 (70%)

Abbreviations: CEP. cerebral embolic protection; Cont., control; DM II, diabetes mellitus IIa; Int., intervention; IQR; interquartile range; SD, standard deviation; TIA, transient ischemic attack.

Notes: ^€^ Mean ± SD or median (IQR). ^¥^ Logistic EuroSCOREII. *The intervention in the Sentinel study was divided into two groups: Device Arm and Safety Arm. ^¶^ Includes both stroke and TIA.

The risk of bias assessment was performed by two researchers independently and blindly. If there were discrepancies between the judgments, a third evaluator would be integrated. Version 2 of the Cochrane risk-of-bias tool for randomized trials (RoB 2; free) was used for risk of bias analysis. 7 The quality of the evidence was assessed using the Grading of Recommendations Assessment, Development, and Evaluation (GRADE) software GRADEpro (Evidence Prime). 8

### Outcomes

The primary outcome assessed was all strokes. Secondary outcomes were divided into safety (all-cause mortality and vascular complications) and efficacy (disabling stroke and TIA) outcomes. Primary and secondary outcomes were assessed within 30 days, except for the British Heart Foundation's (BHF) PROTECT-TAVI study, which reported the occurrence of disabling stroke up to 8 weeks after valve implantation.

### Statistical analysis

Odds ratios (ORs) and 95%CIs were calculated for all outcomes analyzed. Heterogeneity among studies was assessed using the *p* -value of Cochran's Q test statistic, indicating divergence if *p* was ≤ 0.1. In this context, inconsistency was evaluated using the I ^2^ statistic.

All outcomes were analyzed according to random effects modeling using the inverse variance method due to clinical heterogeneity among studies in the approach to different types of CEP. In the sensitivity analysis, the fixed modeling model was changed according to the Mantel–Haenszel method; furthermore, the BHF PROTECT-TAVI study was excluded from the analyses because of the different follow-up time.

This process was performed using the Review Manager (RevMan) statistical software (The Cochrane Collaboration), version 5.4, and trial sequential analysis (TSA, Copenhagen Trial Unit). 9 Given the anticipated clinical heterogeneity, a subgroup analysis was performed according to the type of CEP (filter or deflector) and the access route during TAVI.

The TSA tests was performed for the primary and secondary outcomes to assess the conventional meta-analysis estimates for type-I and -II errors. The required sample size (rSS) for the intervention effect size was calculated considering a type-I error of 5% and a power of 90%, according to the α expenditure model using the O'Brien-Fleming method. For sensitivity analysis, a type-I error of 5% and a power of 99% were assumed. Effect sizes were evaluated sequentially until all available trials were used. The 95%CI was adjusted for type-I and -II errors using the α-spending approach.

Diversity (D ^2^ ) is a meta-analytic parameter calculated by the TSA program that serves as a measure for comparing statistical weights between random effects model methodologies (DerSimonian-Laird, Sidik-Jonkman, and Biggersaff-Tweedie). The cumulative Z-score was calculated to examine the benefit, harm, or futility of the intervention according to its relative position between the intervention, futility line, conventional limit, superiority limit, and rSS. These categories are shown in **Supplementary Material 1 Figure S1** .

A two-tailed significance level of 5% was adopted for significance testing. Considering more clinically realistic estimates of relative risk reduction (RRR), a sensitivity analysis was performed on the TSA for the “all-cause mortality” stages. These analyses were performed using the TSA software. 9

## RESULTS

During the search stage, 744 articles were identified in the consulted databases, according to the methodology. Of this total, 230 articles were removed during duplicate detection, resulting in 514 articles for the screening phase. Based on the eligibility criteria, 506 studies were excluded, resulting in 8 articles being included in the final analysis, as represented in Figure 1 of the PRISMA flowchart. 2 10 11 12 13 14 15 16 Cohen's kappa index was 0.94 (95%CI: 0.82–0.94, *p* < 0.01) representing a strong level of agreement among researchers (data reliability: 82–100%).

**Figure 1:**
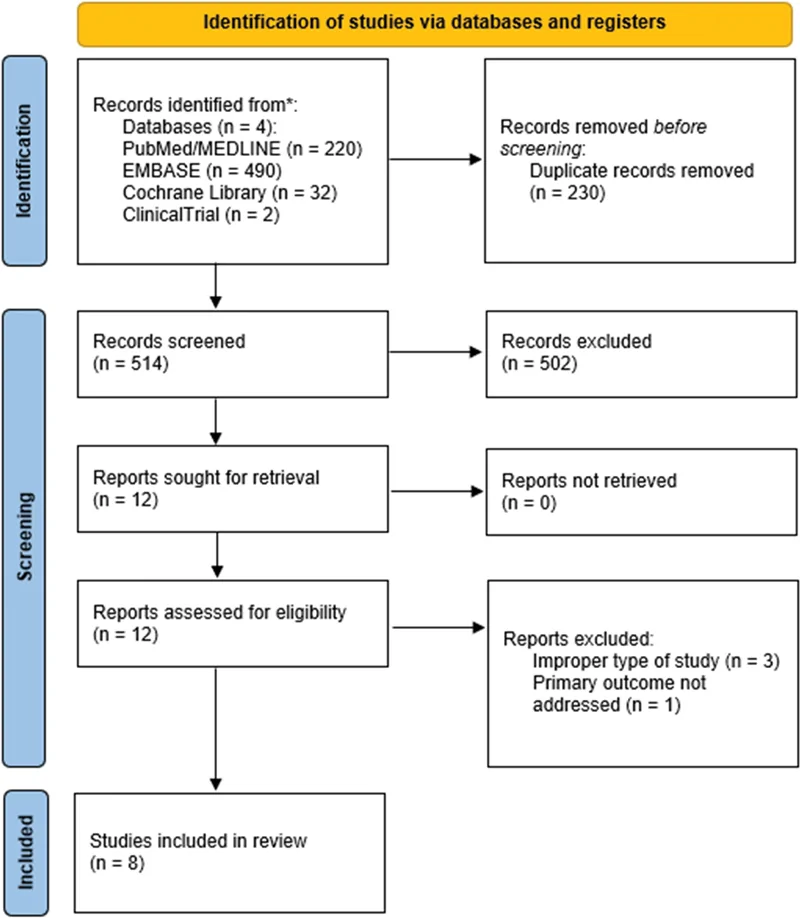
Preferred Reporting Items for Systematic Reviews and Meta-Analyses (PRISMA) flowchart for study selection.

### Risk of bias assessment

The risk of bias assessment for the included studies is shown in ( **Supplementary Material 1 Figure S2** ). In general, the studies presented good methodological quality; however, there are some concerns regarding the patient randomization process, specifically the lack of complementary explanation about how allocation and blinding occurred, and the analysis of results, which did not report complementary justifications for the loss of data from some patients. On the other hand, there was a low risk of bias in the domains of “deviations from planned interventions,” “measurement of the outcome,” and “selection of the reported result.” The Claret Embolic Protection and TAVI (CLEAN-TAVI) and BHF PROTECT-TAVI studies presented an overall low risk of bias, while the others showed some concerns.

According to the GRADE framework, the certainty of evidence was moderate for all strokes, disabling stroke, TIA, and all-cause mortality, and low for vascular complication events ( **Supplementary Material 1 Table S2** ).

### Analysis of primary results

The results of the conventional meta-analysis and the sequential analysis of clinical trials are summarized in Table 2 and illustrated in Figures 2,3 and **Supplementary Material 1 Figures 3–5** . All eight studies presented data on the primary stage of interest ( Figure 2 ). There was no statistically significant difference between the intervention (CEP + TAVI group) and the control (TAVI group) in the occurrence of all types of stroke evidenced in both the meta-analysis and the TSA (OR = 0.92, 95%CI [0.73–1.15], IC _ajusα_ [0.58–1.42], p-value = 0.46), however, the data point to a potential benefit. The rSS for a 10.53% RRR was not reached (n = 11,589 for rSS = 38,626; 30%). The cumulative Z-score was below the conventional and superiority thresholds, and did not reach the generated futility line ( Figure 2 ).

**Table 2:** Meta-analysis and sequential analysis of the results of the included clinical trials

Outcomes	Meta-analysis				TSA						
	OR (CI _95%_ )	*p-value*	* I ^2^* (%)	OR (α _adj_ CI) ^¥^	IIA (%)	ICA (%)	RRR ^¶^	D ^2^ (%)	SS	SS reached	Evidence
All stroke	0.92 (0.73–1.15)	0.46	0	0.9 (0.58–1.42)	7.65	8.55	10.53	0	38,626	No	AE
Disabling stroke	0.80 (0.57–1.13)	0.20	0	0.80 (0.34–1.86)	1.8	2.17	17.05	0	59,732	No	AE
TIA	1.22 (0.63–2.33)	0.56	0	1.22 (0.35–4.19)	0.22	0.42	47.62	0	33,517	No	AE
All-cause mortality	1.09 (0.71–1.69)	0.69	0	1.09 (0.59–2.01)	1.49	2.58	42.25	0	7,053	Yes	Futility
Vascular complication	1.13 (0.74–1.71)	0.57	30	N/A	14.17	13	-9	86	254,055	N/A	AE*

Abbreviations: AE, indicates absent evidence; D2, diversity; I2, Higgins heterogeneity index; ICA, incidence in control arm; IIA, incidence in intervention arm; OR, odds ratio; RRR, relative risk reduction; SS, sample size; TIA, transient ischemic attack; TSA, trial sequential analysis; CIαadj, adjusted confidence interval for spending α.

Notes: *Not available due to insufficient information use. ^¥^ Random effects modeling was used according to the DerSimonian-Laird method. ^¶^ Relative risk reduction defined by the basic formula according to Leviton's notation.

**Figure 2:**
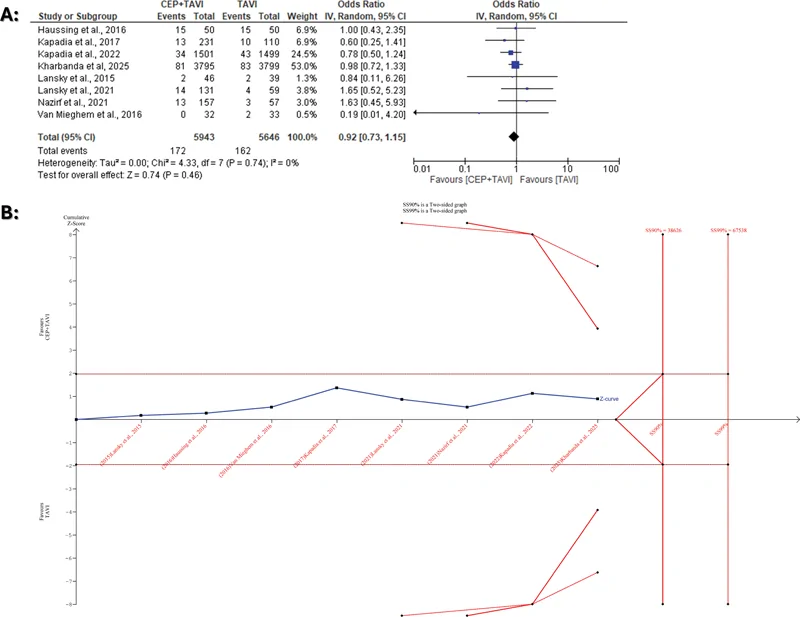
Abbreviations: IV, inverse variance; SS, sample size; TSA, trial sequential analysis. Forest plot and TSA for all strokes.

### Analysis of secondary outcomes

No significant differences were found between the intervention and control groups regarding the occurrence of disabling stroke ( Figure 3 ), as the quantitative synthesis analysis of the included clinical trials identified no statistically significant difference (OR = 0.80; 95%CI: 0.57–1.13; CI _ajusα_ : 0.34–1.86; *p* = 0.20). The sequential analysis of the trials revealed an insufficient sample size to cross the TSA thresholds, meaning additional studies and patients are still needed to draw concise evidence for a RRR of 17.05% (11,589 patients included for an RSS of 59,732, 19.4%).

**Figure 3:**
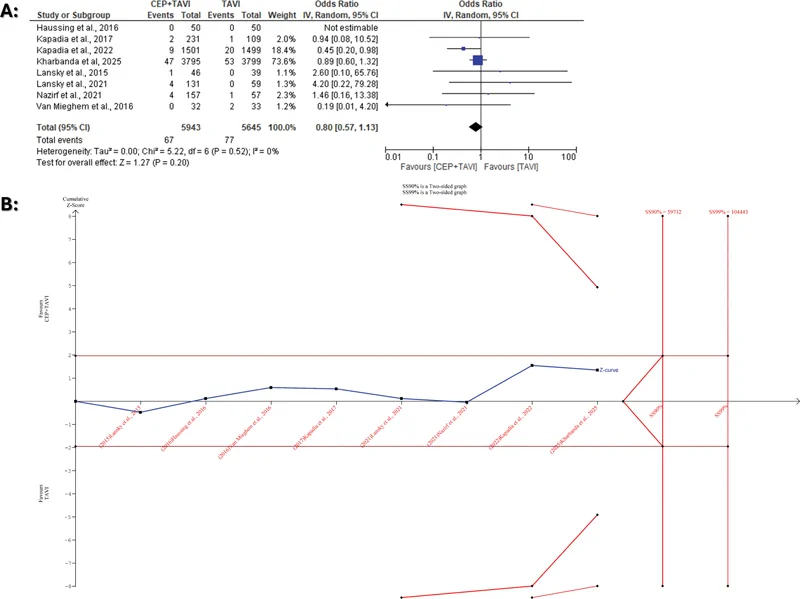
Abbreviations: IV, inverse variance; SS, sample size; TSA, trial sequential analysis. Forest plot and TSA for disabling stroke.

Regarding the occurrence of TIA, only the CLEAN-TAVI, SENTINEL, PROTECTED-TAVI, BHF PROTECT-TAVI, and REFLECT I studies addressed the analysis of this outcome ( Figure 4 ). Conventional meta-analysis identified a statistically insignificant difference between the group that used embolic protection and those that did not (OR = 1.22; 95%CI: 0.63–2.33;, CI _ajusα_ : 0.35–4.19; *p* = 0.56). Sequential analysis identified an insufficient sample size that crossed the limit generated by the TSA. Therefore, an additional number of patients is required to enable solid evidence for a RRR of 47.62% (n = 11,589 for an rSS = 33,517, 34.6%). The cumulative Z-score remained below the superiority threshold and the conventional threshold, not crossing the futility line.

**Figure 4:**
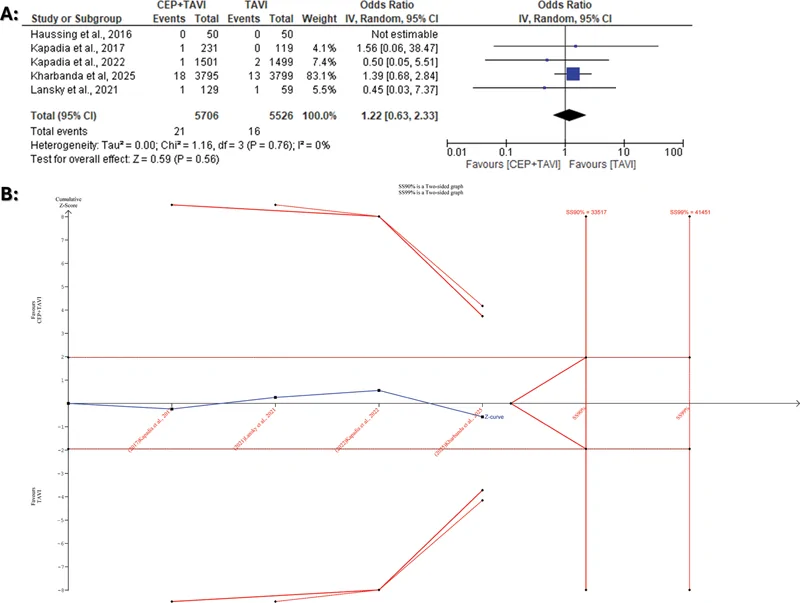
Abbreviations: IV, inverse variance; SS, sample size; TIA, transient ischemic attacks; TSA, trial sequential analysis. Forest plot and TSA for TIA.

### Analysis of safety outcomes

For safety outcomes, all-cause mortality showed no statistically significant difference between the CEP and non-CEP groups in both conventional meta-analysis and TSA (OR = 1.09, 95%CI: 0.71–1.69; CI _ajusα_ : 0.59–2.01; *p* = 0.69), as shown in Figure 5 . The cumulative Z-score remained below the conventional superiority line, entered the futility area, and exceeded the TSA limit for the sample size (n = 11,589 patients, included for an rSS = 7,053). This result means that there is evidence to support the futility of using or not using an embolic protection device to reduce the relative risk of mortality by 42.25%. Sensitivity analysis considering incidence rates from studies with large sample sizes estimated a more realistic RRR of 25.6%. However, no change was identified in the futility hypothesis for the previously found outcome ( **Supplementary Material 2** – available at https://www.arquivosdeneuropsiquiatria.org/wp-content/uploads/2026/07/ANP-2025.0503-Supplementary-Material-2.docx ). 17

**Figure 5:**
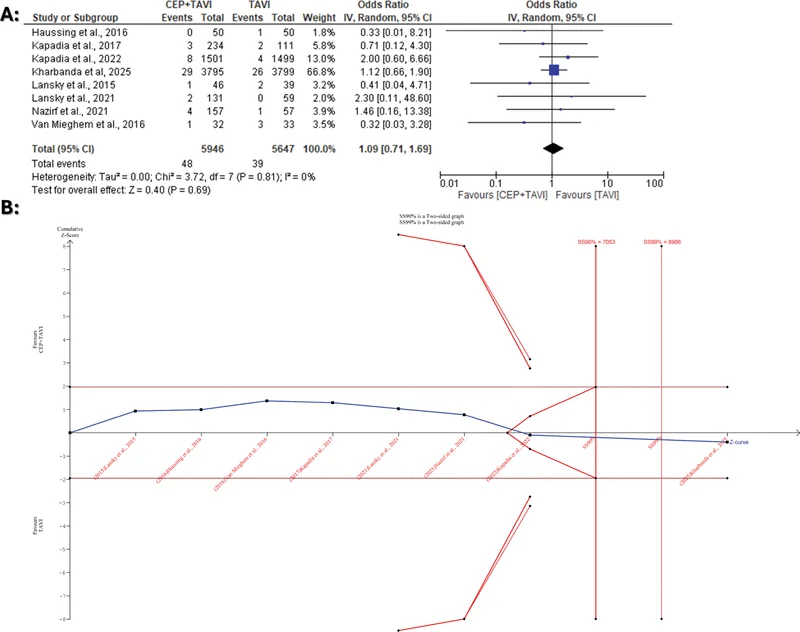
Abbreviations: IV, inverse variance; SS, sample size; TSA, trial sequential analysis. Forest plot and TSA for all-cause mortality.

For vascular complications, no statistically significant difference was found between the intervention and control groups in the occurrence of vascular complications (OR = 1.13; 95%CI: 0.74–1.71; *p* = 0.57), as presented in the **Supplementary Material 1 Figure S3** . The rSS was insufficient to generate analysis by TSA, as it did not reach the two-tailed threshold of 5% (11,589 patients, included for a required size of 254,055, 4.5%). Exploring the heterogeneity of the outcome, it was remedied by excluding the REFLECT I and II studies from the quantitative synthesis due to the discrepancy in effect size compared with the others; even so, there was insignificance between the groups (OR = 1.04, 95%CI: 0.89–1.22, *p* = 0.61). This is possibly due to a difference in the randomization of patients, since in these two studies there was an allocation in the proportion between the intervention and control group of 2:1 concomitantly with a roll-in process without randomization.

Sensitivity analysis for all outcomes using 99% power demonstrated similar results, as did sensitivity analysis when the effects modeling was changed to fixed according to the Mantel-Haenszel method ( **Supplementary Material 1 Table S3** ). Exclusion of the BHF PROTECT-TAVI study did not materially alter the findings of the conventional meta-analysis. However, in the TSA, changes were observed for specific outcomes. For TIAs, the interpretation shifted from a low probability of significant benefit due to insufficient evidence, to confirm or refute effects of the intervention. Conversely, for all-cause mortality, the interpretation changed from futility of the intervention to a low probability of significant benefit due to insufficient evidence ( **Supplementary Material 3** – available at https://www.arquivosdeneuropsiquiatria.org/wp-content/uploads/2026/07/ANP-2025.0503-Supplementary-Material-3.docx ).

### Subgroup analysis

The analysis considering the types of CEP's (filter: Sentinel and Claret Medical; deflector: TriGuard) did not indicate a difference between the subgroups and in relation to the summarized effect of the CEPs for all outcomes analyzed ( **Supplementary Material 1 Figure S4** ). Stratification of studies by TAVI access route yielded consistent results with the overall analysis ( **Supplementary Material 1 Figure S5** ).

## DISCUSSION

The introduction of CEP as a prophylactic measure for stroke and other cerebrovascular outcomes in TAVI transplants is based on the pathophysiological basis of thrombus formation resulting from valve replacement, acting through a mechanism that filters these elements before they reach the cerebral circulation. This effort to reduce the occurrence of strokes in these procedures, in addition to reducing perioperative morbidity and mortality, also aims to reduce hospital costs, including neurosurgical procedures, hospital stays, and rehabilitation support, and improve the quality of life for these patients.

The present study presented remarkable results when analyzing the use of these devices. Primarily, for the outcomes of all strokes, those classified as disabling, and TIAs, the conventional meta-analysis indicated no statistically significant difference of CEP use compared with the placebo group. However, the sample size collected was insufficient to obtain conclusive evidence based on the calculated TSA. For all strokes and disabling strokes, the relative effect size along with the confidence interval reveals a potential reduction with CEP use and a potential increase in TIA with these devices.

The ORs for all strokes within 30 days (OR: 0.91, 95%CI: 0.73–1.15; *p* = 0.46) and disabling stroke within 30 days (OR: 0.80, 95%CI: 0.57–1.13; *p* = 0.20) were not statistically significant between the intervention and control groups; however, they suggest a potential clinical benefit associated with the use of embolic protection devices, particularly with the inclusion of additional data.

When incorporating TSA, the cumulative Z-curves lie within the zone favoring the intervention, yet remain within the boundaries of statistical equivalence, reinforcing the absence of a definitive difference while indicating a trend toward benefit. Notably, the cumulative Z-curve does not cross the futility boundary, the required information size, or the superiority boundary, indicating that TSA provides only weak evidence to clarify the true effect of CEP. Overall, the evidence remains inconclusive but is consistent with a potential clinical benefit, reflecting uncertainty regarding a clinically meaningful effect. Therefore, further adequately powered randomized clinical trials are warranted to better define the clinical impact of this intervention.

Secondarily, futility and the required sample size were achieved in the analysis of all-cause mortality, revealing sufficient evidence that the use of CEP or not had no statistically significant difference on the outcome assessed. Regarding vascular complications, there was no difference between the groups. However, it was not possible to extract sufficient evidence due to the small sample size collected for this outcome. Therefore, there does not appear to be a clear association between the use of the intervention and resulting complications.

In the subgroup analysis, no statistically significant differences were observed based on device type (filter versus deflector) or TAVI access route. No differences were identified either between subgroups or between intervention and control groups within subgroups for the outcomes analyzed. These findings suggest that the anticipated clinical heterogeneity does not meaningfully influence the potential effect of embolic protection devices in reducing all strokes or disabling strokes. Accordingly, the choice of CEP type or implantation route does not appear to modify clinical outcomes. Therefore, other considerations—such as device cost, technical expertise, and patient-specific anatomical factors—should guide clinical decision-making.

Excluding the BHF PROTECT-TAVI study did not materially affect pooled estimates. The TSA results indicated futility for TIA and insufficient evidence for benefit in all-cause mortality. These findings do not alter the clinical interpretation of the conventional analysis, suggesting minimal impact of follow-up heterogeneity on effect estimates.

In the risk-of-bias assessment, some concerns were identified regarding the randomization process, as several included studies ( **Supplementary Material 1 Figure S2** ) did not provide sufficient details on allocation concealment and blinding procedures. This lack of methodological transparency may undermine confidence in the individual study findings and could potentially lead to an overestimation of the benefits of CEP, thereby distorting the reported incidence of cerebrovascular outcomes and inflating the overall effect estimates.

Although the risk of bias was formally assessed using the RoB 2 tool, a more comprehensive consideration of its potential impact is warranted. While most domains were judged as low risk, some concerns were identified in the randomization process (domain 1) for five studies, and in missing outcome data dDomain 3) for three. These limitations may introduce selection and attrition bias, potentially resulting in systematic differences between groups or incomplete outcome ascertainment, thereby influencing both the magnitude and direction of pooled estimates.

In this context, the overall certainty of the evidence, as evaluated by the GRADE framework, was rated as moderate for most outcomes and low for vascular complications, primarily due to concerns related to risk of bias and imprecision driven by low event rates. Taken together, these findings suggest that, although the included studies are predominantly randomized, the presence of methodological limitations reduces confidence in the estimated effects. This should be carefully considered when interpreting the results, particularly in light of trial sequential analysis, which indicates that the current body of evidence remains insufficient to support definitive conclusions.

Although CEP devices are designed to reduce embolic burden during TAVI, current evidence does not demonstrate a consistent reduction in clinically relevant neurological events. Their routine use is associated with increased procedural costs, and given the low absolute incidence of stroke, their cost-effectiveness in an unselected TAVI population remains uncertain. Their economic value may be more favorable in populations with a higher baseline risk of neurological events. 18

Although no clear benefit was observed in the overall population, certain high-risk subgroups—including patients with prior stroke, atrial fibrillation, or extensive aortic atheroma—may experience greater absolute benefit from embolic protection devices, given their elevated baseline embolic risk and heightened vulnerability to procedure-related cerebral embolization. Further studies specifically targeting these populations are needed to better delineate the clinical utility of this strategy.

Ongoing and future randomized controlled trials with larger sample sizes and longer follow-up are essential to clarify the clinical impact of embolic protection devices. Particular emphasis should be placed on patient-centered outcomes, including disabling stroke and long-term neurological function. Recent ongoing and pending studies may contribute to redefining the role of CEP devices during TAVI.

The SHIELD trial (NCT06689839) evaluates a next-generation filter system, potentially improving embolic debris capture. 19 The PROTEMBO IDE trial (NCT05873816) investigates a device designed to provide broader coverage of cerebral arterial territories, aiming to overcome limitations observed with earlier devices. 20 Meanwhile, the PROTECT H2H trial (NCT05684146), with completed enrollment and results still pending, proposes an innovative approach with circumferential protection, including both cerebral and systemic territories. 21 Taken together, these studies reflect a shift on focus from evaluating embolic protection as a class effect to assessing next-generation technologies with potential impact on the prevention of neurological events.

Current evidence suggests that while embolic protection devices are biologically plausible, their clinical and economic impact in routine practice remains uncertain. A more selective, risk-based approach may represent a more appropriate strategy until further high-quality evidence becomes available.

In the literature, there is a discrepancy between the conclusions and results of previous meta-analyses regarding the inclusion of nonrandomized and randomized studies in the same analysis. Previous studies that included both types of studies identified a reduction in all strokes with the use of embolic protection devices. 22 23 However, more recent meta-analyses that included only randomized clinical trials with the same results presented here. 3 24

This difference in results may stem from methodological differences between the two study subtypes, which may mask the benefit of using CEP in reducing cerebrovascular events analyzed in randomized studies compared with its routine application in cohort studies, such as a retrospective cohort study of 414,649 patients that reported a reduction in the occurrence of disabling stroke in individuals who used embolic protection. 25 Furthermore, the power related to the sample size of patients included may also influence this difference between the results in the literature, corroborated by the TSA.

The likely potential of using CEP in TAVI was also addressed in a meta-analysis and Bayesian approach, which highlighted a high probability of beneficial effects for patients in preventing disabling stroke, but the intervention was unlikely to be clinically relevant. 24

A recent meta-analysis and TSA presented similar findings to this study for most of the outcomes analyzed, with the exception of TIAs. This study does not include the BHF PROTECT-TAVI study, which included 7,635 patients and had a large sample size, sufficient to support the insignificance of the intervention in relation to all-cause mortality previously reported. However, it has not yet generated sufficient evidence for meta-analytic conclusions for the other outcomes. 3 Therefore, additional data from new clinical trials are needed.

Different statistical approaches, such as TSA and Bayesian analysis, are well-known for generating more robust evidence that complements conventional meta-analysis, given the limitations of relying solely on the latter method. From this perspective, it is worth highlighting the auxiliary power of TSA and the primary importance of meta-analysis in obtaining the findings of effect summarization. The Cochrane Scientific Committee recommends that meta-analytic evidence be interpreted based on its magnitude and uncertainty.

### Study limitations

The limitations of this meta-analysis are not limited to study designs, but also relate to the operational variations inherent to any procedure and the type of protective device used.

Among the design limitations, the 72-hour follow-up period for assessing cerebrovascular events, adopted by some of the included trials, may obscure the same outcomes that occurred at a later time interval. However, this limitation is mitigated since all studies included stroke investigation within 30 days of the procedure in their designs, and the sensitivity analysis did not identify a difference in effects. It was not possible to perform a sensitivity analysis considering a more likely RRR for the occurrence of TIA due to a lack of realistic incidence data in real-world studies.

Regarding operational limitations, it is noteworthy that, although surgical techniques vary little between procedures, the surgeons are always different, leading to varying results and increased procedure time. This limitation becomes even more challenging in studies that included complex anatomical cases, exemplified by excessive twisting of the brachiocephalic artery or an elongated left common carotid artery, among other variations. However, with increased operator experience, an overall reduction in time was observed over the course of the surgeries. 10 A subgroup analysis by a high-risk population was not possible due to a lack of pooled data for each classification of past medical history, since the included clinical trials did not present this data in their full articles, supplementary material, or post hoc analysis.

Regarding limitations related to the device, it is important to note that the filters used were of different models, divided into two main types: SENTINEL and TRIGUARD. The first has a filter mechanism, while the second has a deflector mechanism, characteristics that may prevent acurate comparison of efficacy between studies; however, no differences in effects were reported considering subgroup analysis.

Finally, in the SENTINEL trials, the anatomical territory supplied by the left vertebral artery was left unprotected due to the device's limitations, a factor that may have contributed to the greater number of negative cerebrovascular outcomes for patients.

In conclusion the conventional random-effects modeling meta-analysis demonstrated statistical insignificance of CEP devices in reducing the odds of all strokes, disabling strokes, TIAs, all-cause mortality, and vascular complications. The TSA results determined that for all-cause mortality, the sample size was sufficient to generate evidence supporting the futility of embolic protection devices. However, for the other outcomes, the sample size was insufficient to provide conclusive evidence of the meta-analysis findings. Therefore, further trials with similar designs and larger populations are needed to demonstrate the reproducibility of results in different groups and to mitigate the limitations already detailed.
